# Rupiah exchange rate prediction against the US Dollar using a deep neural network with a multi-output sliding window approach

**DOI:** 10.1016/j.mex.2025.103692

**Published:** 2025-10-30

**Authors:** Ihsan Fathoni Amri, Novia Yunanita, Febi Anggun Lestari, Oktaviana Rahma Dhani

**Affiliations:** Department of Data Science, Universitas Muhammadiyah Semarang, Jl. Kedungmundu Raya No.18, Semarang 50273, Indonesia

**Keywords:** Forecasting, GRU, Multi-output regression, Sliding window, Deep learning

## Abstract

The Rupiah USD exchange rate is a critical macroeconomic indicator in Indonesia, yet its prediction remains challenging due to volatility, nonlinear dynamics, and seasonal fluctuations. This study proposes a deep learning-based forecasting approach using a multi-output sliding window framework to simultaneously predict buying and selling rates. Daily historical data from 2015 to 2025 were normalized and enhanced with sine–cosine time encodings to capture weekly cyclical patterns. Three neural network architectures, Multi-Layer Perceptron (MLP), Long Short-Term Memory (LSTM), and Gated Recurrent Unit (GRU) were evaluated, with Vector Autoregression (VAR) serving as a statistical benchmark. Model performance was assessed using MAPE, RMSE, and R². The GRU model demonstrated superior accuracy and stability across volatile periods. A secondary evaluation with a 70:30 train test split confirmed its robustness, achieving an RMSE of 64.57, MAPE of 0.0031, and R² of 0.9875. It also produced reliable short-term forecasts up to seven days ahead, underscoring its practical applicability for financial decision making.

• The method enables simultaneous prediction of buying and selling rates using a multi-output sliding window.
• GRU showed the highest accuracy compared to LSTM, MLP, and VAR, with consistent performance across different splits.
• The approach supports short-term economic forecasting and decision-making in volatile currency environments.

## Graphical abstract

**Figure:**
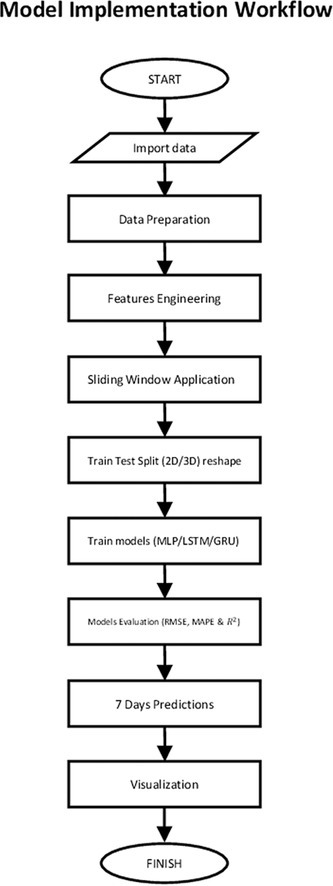

## Specifications table

**Table:** 

**Subject area**	Computer Science
**More specific subject area**	Time Series Forecasting using Deep Learning
**Name of your method**	Deep Neural Network with Multi-Output Sliding Window for Exchange Rate Prediction
**Name and reference of original method**	S. Gao et al., "Short-term runoff prediction with GRU and LSTM networks without requiring time step optimization during sample generation," J. Hydrol., vol. 589, p. 125,188, 2020, doi: https://doi.org/10.1016/j.jhydrol.2020.125188.
**Resource availability**	Dataset: Bank Indonesia Exchange Rates – https://www.bi.go.id/id/statistik/informasi-kurs/transaksi-bi/default.aspx Implementation code available upon request from the corresponding author.

## Background

The exchange rate of the Indonesian Rupiah against the United States Dollar (USD/IDR) is one of Indonesia’s fundamental macroeconomic indicators. Its fluctuations directly affect price stability and inflation, serve as a key consideration in the formulation of monetary policy by Bank Indonesia, and determine the level of risk and profitability for businesses and investors [1]. The highly volatile and non-stationary nature of the foreign exchange market makes predicting this currency pair a significant challenge in time series forecasting [2]. This challenge has driven the continuous development of methodologies capable of capturing the market’s complex and rapidly changing dynamics.

Historically, traditional statistical approaches such as the Autoregressive Integrated Moving Average (ARIMA) and Generalized Autoregressive Conditional Heteroskedasticity (GARCH) models have been widely used as references in financial time series modeling [3,4]. While effective in capturing linear patterns and volatility clustering, these models have inherent limitations in identifying nonlinear and complex temporal dependencies[5]. Such limitations become increasingly evident when dealing with market data influenced by multiple nonlinear external factors, where traditional models often fail to provide adequate accuracy[6]. With advances in computing power, deep learning technology has emerged as a far more adaptive and powerful alternative, consistently outperforming traditional models in various recent financial forecasting studies[7].

Modern research focuses primarily on deep learning architectures that have demonstrated their superiority. The Multilayer Perceptron (MLP) offers strong universal function approximation capabilities, theoretically allowing it to approximate any function with a desired degree of accuracy[8]. Meanwhile, the Long Short-Term Memory (LSTM) and Gated Recurrent Unit (GRU) architectures are specifically designed with gating mechanisms for long-term memory, making them highly effective for processing sequential data and retaining information over time[9,10]. Recent studies explicitly show that LSTM- and GRU-based models significantly outperform ARIMA in exchange rate forecasting accuracy due to their ability to capture complex nonlinear patterns[11]. Even though more advanced architectures such as Transformers are beginning to be adopted, LSTM and GRU remain powerful and efficient choices for many forecasting applications[12]. Moreover, recent studies indicate that GRU often delivers performance comparable to LSTM but with greater computational efficiency, making it an attractive option for real-time forecasting[13].

To evaluate the superior performance of modern neural network models, this study also employs conventional statistical models as benchmarks. The Vector Autoregression (VAR) model is selected as the baseline comparison due to its ability to model linear interdependencies among multiple time series simultaneously, making it a relevant benchmark for multi-output models[14].

Despite these advances, most exchange rate forecasting studies still exhibit research gaps. Many focus solely on predicting a single price type (e.g., selling rate) while ignoring the buying rate, thereby overlooking the valuable information contained in the bid-ask spread—the difference between buying and selling prices, which serves as an indicator of market liquidity and transaction costs[15]. A recent literature review confirms that univariate approaches still dominate this field, even though multivariate approaches that simultaneously consider both prices can provide richer insights[16]. Recent studies have developed multi-output machine learning systems specifically optimized for financial time series forecasting, demonstrating significant improvements in accuracy[17]. Furthermore, calendar effects, such as the “day-of-week effect,” which has been shown to influence exchange rate fluctuations, are rarely incorporated explicitly into predictive model architectures[15]. This effect has been found to impact exchange rate volatility in several markets, including emerging markets such as India, yet it remains underexplored in most predictive models[18].

Based on these research gaps, this study defines several specific problem boundaries. First, it explicitly focuses on the simultaneous prediction of buying and selling USD/IDR rates, directly addressing the limitations of univariate approaches that ignore the spread. Second, the developed model integrates cyclical time features (e.g., day of the week) as exogenous variables to capture weekly seasonal patterns. Third, the study restricts performance comparisons to three modern neural network architectures (MLP, LSTM, GRU), using a conventional statistical model (VAR) as the benchmark. Thus, this research does not aim to test all existing models but rather to provide a measurable methodological contribution to exchange rate forecasting through a multi-output approach using state-of-the-art models.

The methodology proposed in this article seeks to bridge the identified research gaps by implementing a multi-output sliding window approach. This approach enables the simultaneous prediction of buying and selling USD/IDR rates within a single model architecture while integrating cyclical time features to capture weekly seasonal patterns. This configuration is expected to provide a more comprehensive and accurate representation of exchange rate behavior, which can subsequently be replicated and evaluated by other researchers in financial forecasting tasks involving non-stationary and nonlinear time series data.

## Method details

In the study of financial markets, accurately anticipating currency movements is critical for policymakers, investors, and businesses engaged in international trade. Predicting such movements requires careful analysis of historical trends while accounting for market volatility and nonlinear dynamics. Forecasting refers to the process of estimating future values based on historical patterns observed in past data [19]. In this study, forecasting is applied to predict both the selling and buying rates of the Indonesian Rupiah (IDR) against the United States Dollar (USD) using a time series modeling framework [20]. Traditional statistical models such as ARIMA have been widely used for such tasks, but they exhibit limitations in addressing the nonlinearities and complex dynamics characteristic of financial data [21]. To overcome these challenges, this methodology adopts deep learning-based techniques capable of capturing intricate temporal dependencies without relying solely on linear assumptions.

### Data preparation and features engineering

The data used in this study comes from the daily exchange rate report published by Bank Indonesia, covering two main variables, namely the selling rate and the buying rate against foreign currencies, with a period of 2,559 lines from January 2015 to June 2025. The original data is displayed as follows, as shown in Table 1.

**Table 1:** Rupiah exchange rate against US Dollar.

Index	Value	Selling Rate	Purchase Rate	Date
0	1	16,314	16,152	6/30/2025 12:00:00 AM
1	1	16,373	16,211	6/26/2025 12:00:00 AM
…	…	…	…	…
2557	1	12,652	12,526	1/5/2015 12:00:00 AM
2558	1	12,536	12,412	1/2/2015 12:00:00 AM

The initial step of data cleansing is carried out by converting the exchange rate from the string data type to a float numerical format to match the mathematical operation and the needs of the modeling algorithm. The date format is also changed to a datetime type to ensure that each entry is temporally recognizable. After that, the data is rearranged based on the 'Date' column to maintain a chronological order, according to the nature of the time series data which is an important prerequisite in the application of temporal-based predictive models such as LSTM and GRU.

To improve the model's ability to capture temporal seasonality, the feature engineering process incorporates cyclical time components representing the day of the week, transformed into paired sine and cosine values using the cyclical Fourier approach. With the formula:

$$sin\_day=\;sin\left(\frac{\left(2\pi \;\cdot \;dayofweek\right)}{7}\;\right),cos\_day\;=\cos\left(\frac{\left(2\pi \;\cdot \;dayofweek\right)}{7}\;\right)$$

This transformation preserves the proximity of days such as Monday and Sunday despite their ordinal separation, maintaining seasonal continuity and helping models like MLP, LSTM, and GRU identify weekly recurring patterns in exchange rate data [22]. An example of the results of adding this feature is shown in the form of the following table (see Table 2).

**Table 2:** Additional features engineering.

Index	Selling Rate	Purchase Rate	sin_day	cos_day
0	12,536.00	12,412.00	−0.433884	−0.900969
1	12,652.00	12,526.00	0.000000	1.000.000
…	…	…	…	…
2557	16,373.46	16,210.54	0.433884	−0.900969
2558	16,314.17	16,151.83	0.000000	1.000.000

Furthermore, all features, including sell rate, buy rate, sin_day, and cos_day, are normalized into a range of 0 to 1 using the Min-Max Scaling technique, as shown in Table 3. This normalization is necessary to equalize the scale between features so that the model does not have a bias towards features with large numerical values.

**Table 3:** Normalization of features.

Index	Selling Rate	Purchase Rate	sin_day	cos_day
0	0.006635	0.006702	0.277479	0.000000
1	0.032289	0.032170	0.500000	1.000.000
…	…	…	…	…
2557	0.855307	0.855295	0.722521	0.000000
2558	0.842195	0.842179	0.500000	1.000.000

### Sliding window application

The sliding window transformation is an essential step to restructure sequential time series data into a supervised learning format, enabling deep learning models such as MLP, LSTM, and GRU, none of which inherently interpret temporal order to process the information effectively [23]. In this study, a 7-day rolling window is employed to align with the weekly cycle of financial market activity. Through this method, sequences of the previous seven consecutive days, each comprising the selling rate, buying rate, and the cyclical sine–cosine time features obtained from the feature engineering stage [22], are designated as the input set (*X* = {xₜ₋₇, xₜ₋₆, …, xₜ₋₁}), while the corresponding selling and buying rates of the following day serve as the target output (*Y* = {yₜ}). This structure allows the model to systematically learn short-term temporal patterns and seasonal effects embedded in the data. Once the sliding window is applied, the datasets are then organized into two specific formats: (1) a two-dimensional array for MLP models, treating each sequence as a flat feature vector, and (2) a three-dimensional array [samples, timesteps, features] for sequence-oriented architectures like LSTM and GRU, thereby enabling these models to capture and leverage temporal dependencies with higher fidelity.

### Training and testing strategy

The dataset is divided into 80 % for training and 20 % for testing, without applying any random shuffling, to preserve the chronological sequence essential for time series forecasting[24]. This sequential integrity ensures that the models MLP, LSTM, and GRU are trained exclusively on historical data and evaluated on future, unseen observations, thereby safeguarding temporal causality and enabling a realistic assessment of predictive performance. Furthermore, the input data is structured according to the specific requirements of each model: a two-dimensional format (samples × features) for MLP, and a three-dimensional format (samples × timesteps × features) for LSTM and GRU. This tailored formatting enables MLP to process flattened feature vectors effectively, while allowing LSTM and GRU to capture temporal dependencies more accurately, optimizing their ability to model sequential patterns in the exchange rate data.

### Multi-output regression

The multi-output regression approach is used so that the model can predict two targets at once, namely the selling and buying rates of the Rupiah against the US Dollar, consistently and efficiently. This method allows modeling of internal relationships between targets [25], thus maximizing the information that the model can learn in a single training process.

### Model architectures

1) Multi-Layer Perceptron (MLP)

The Multi-Layer Perceptron (MLP) is a feedforward neural network composed of an input layer, one or more hidden layers, and an output layer [26]. In each hidden layer, neurons apply a linear transformation to their inputs, add a bias, and pass the result through a nonlinear activation such as ReLU or sigmoid [27]. This enables the model to learn non-linear patterns between features [28]. Its general regression form can be written as:

$$\hat{y}=f\left(W_{n}\cdot \sigma \left(W_{n-1}\cdot \ldots \sigma \left(W_{1}\cdot x+b_{1}\right)+\ldots +b_{n-1}\right)+b_{n}\right)$$

where $W_{i}\;$and $b_{i}\;$are the weight and bias of the i-th layer, σ is the activation, and is x the input. As MLPs have no internal memory, time series tasks require preprocessing like sliding windows [23]. Overfitting risks are reduced via dropout and early stopping [29].

In this work, the MLP serves as a baseline, tested in five hidden-layer configurations to explore capacity variations, as detailed in Table 4.

2) Long Short-Term Memory (LSTM)

**Table 4:** MLP model architecture.

Type	1st Layer	2nd Layer	3rd Layer	Output Layer
MLP-Small	Dense(32, ReLU)	Dense(16, ReLU)	–	Dense(1, Linear)
MLP-Medium	Dense(64, ReLU)	Dense(32, ReLU)	–	Dense(1, Linear)
MLP-Large	Dense(128, ReLU)	Dense(64, ReLU)	Dense(32, ReLU)	Dense(1, Linear)
MLP-Tuned1	Dense(128, ReLU)	Dense(64, ReLU)	Dense(32, ReLU)	Dense(1, Linear)
MLP-Tuned2	Dense(256, ReLU)	Dense(128, ReLU)	Dense(64, ReLU)	Dense(1, Linear)

The Long Short-Term Memory (LSTM) network is an advanced Recurrent Neural Network (RNN) architecture explicitly designed to address the vanishing gradient problem and capture long-term dependencies in sequential data [10]. It achieves this through the use of a cell state that acts as an internal memory, and three gating mechanisms—input, forget, and output gates—that control the addition, removal, and exposure of information at each time step. Mathematically, the LSTM operations can be expressed as:

$$\begin{array}{c} f_{t}\;=\;\sigma \left(W_{f}\;\cdot \;\left[h_{t-1},\;x_{t}\right]\;+\;b_{f}\right)\;i_{t}\;=\;\sigma \left(W_{i}\;\cdot \;\left[h_{t-1},\;x_{t}\right]\;+\;b_{i}\right)\;o_{t}\;=\;\sigma \left(W_{o}\;\cdot \;\left[h_{t-1},\;x_{t}\right]\;+\;b_{o}\right)\;{\overset{\wedge }{C}}_{t}\\ =\;tanh\left(W_{c}\;\cdot \;\left[h_{t-1},\;x_{t}\right]\;+\;b_{c}\right)\;C_{t}\;=\;f_{t}\;*\;C_{t-1}\;+\;i_{t}\;*\;\overset{\wedge }{C}\;h_{t}\;=\;o_{t}\;*\;tanh\left(C_{t}\right) \end{array}$$

where *f*_t_ is the forget gate, *i*_t_ the input gate, oₜ the output gate, *C*_t_ the cell state, and hₜ the hidden state. These gates allow the LSTM to retain relevant historical patterns and discard noise, making it highly effective in modeling complex trends and seasonality in financial time series. In this study, the LSTM is implemented with a single recurrent layer of 64 memory units using the Tanh activation to process sequential inputs, followed by a dropout layer with a rate of 0.2 to reduce overfitting. The final Dense output layer consists of two linear units to enable simultaneous multi-output prediction of the selling and buying rates. A summary of the LSTM model architecture used in this study is provided in Table 5.

3) Gated Recurrent Unit (GRU)

**Table 5:** LSTM model architecture.

Layer Type	Number of Units	Activation
LSTM Layer	64	Tanh
Dropout	0.2	–
Dense (Output)	2	Linear

The Gated Recurrent Unit (GRU) is a streamlined variant of the LSTM architecture that consolidates the input and forget gates into a single update gate, and substitutes the separate output modulation process with a reset gate [13]. This simplification not only reduces computational overhead but also preserves the model’s ability to effectively capture and exploit temporal dependencies in sequential data. The GRU’s operations can be mathematically expressed as

$$z_{t}\;=\;\sigma \left(W_{z}\cdot \;\left[{h_{t}}^{-1},\;x_{t}\right]\right)r_{t}=\;\sigma \left(W_{r}\cdot \;\left[{h_{t}}^{-1},\;x_{t}\right]\right)\;{\tilde{h}}_{t}\;=\;tanh\left(W\;\cdot \;\left[r_{t}\;*\;{h_{t}}^{-1},\;x_{t}\right]\right)h_{t}\;=\;\left(1\;-\;z_{t}\right)*\;{h_{t}}^{-1}+\;z_{t}\;*\;{\tilde{h}}_{t}$$

where *z*_t_ controls the update of the hidden state and *r*_t_ determines how much past information to forget. In this study, the GRU is implemented with a single recurrent layer of 64 units using Tanh activation, followed by a Dense output layer with two linear neurons to produce simultaneous forecasts for both the selling and buying exchange rates. A concise overview of the GRU implementation is provided in Table 6.

4)Vector Autoregression (VAR)

**Table 6:** GRU model architecture.

Layer Type	Number of Units	Activation
GRU Layer	64	Tanh
Dense (Output)	2	Linear

The Vector Autoregression (VAR) model is a classical multivariate time series approach designed to capture the linear interdependencies among several variables that evolve over time.

Unlike univariate autoregressive models, which involve only a single variable, VAR treats all variables in the system as endogenous meaning that each variable can depend not only on its own past values but also on the lagged values of other variables within the system[30].

This approach is particularly suitable for analyzing the dynamic relationship between the selling and buying exchange rates within a currency market framework.

The general form of a VAR(p) model with two variables can be expressed as:

$$Y_{t}=\;c\;+\;A_{1}Y_{\left\{t-1\right\}}+\;A_{2}Y_{\left\{t-2\right\}}+\;\ldots +\;A_{p}Y_{\left\{t-p\right\}}+\;\varepsilon_{t}$$

Where $Yt=\;{\left[y1t,y2t\right]}^{T}$ represents a vector of endogenous variables at time ttt; ccc denotes the vector of intercepts; $A_{i}$​​ are the coefficient matrices corresponding to lag i; and $\varepsilon_{t}$​ is a vector of white-noise error terms[31].

The optimal lag length (p) is commonly determined using information criteria such as the Akaike Information Criterion (AIC) or the Bayesian Information Criterion (BIC)[31]. In this study, the VAR model is employed as a statistical benchmark to evaluate the performance of deep learning models (MLP, LSTM, and GRU) in capturing the linear dependencies between the selling and buying rates of the Rupiah against the US Dollar. Further details of the VAR architecture used for comparison are provided in Table 7.

**Table 7:** VAR model architecture.

Layer Type	Number of Units / Lags	Activation / Notes
VAR Layer	2 (endogenous)	Linear (log returns)
Lag Selection	1–5 (dynamic, AIC)	–
Rolling Forecast Window	60 observations	–
Output Transformation	2 (Kurs Jual, Kurs Beli)	Exponentiated from log returns
VAR Layer	2 (endogenous)	Linear (log returns)

### Model evaluation

The performance of each model is assessed using three main evaluation metrics: Mean Absolute Percentage Error (MAPE), Root Mean Squared Error (RMSE), and the Coefficient of Determination (R²).

MAPE measures prediction errors as a percentage of the actual values, providing intuitive interpretability, while R² quantifies the proportion of variance in the dependent variable explained by the model.

RMSE is introduced as an additional metric to measure the average magnitude of prediction errors in the same units as the target variable, penalizing larger errors more heavily than MAPE[32].

The mathematical formulations are defined as follows:

$$MAPE=\left(\frac{100}{n}\right)\sum \frac{yi-\hat{yi}}{yi}$$

$$RMSE=\;\sqrt{\frac{1}{n\;}\;\sum_{i=1}^{n}{\left(yi-\hat{y\;i}\right)}^{2}}$$

$$R^{2\;}=1-(\frac{\sum {\left(yi-\hat{yi}\right)}^{2}}{\sum {\left(yi-\hat{yi}\right)}^{2}}$$

Before calculating these metrics, all predicted values are inverse-transformed using the MinMaxScaler to restore them to the original exchange rate scale, ensuring real-world interpretability.

The inclusion of RMSE complements MAPE and R² by providing a scale-sensitive error measure that captures the model’s predictive precision. This combination of evaluation metrics enables a fair and consistent comparison between the classical VAR benchmark and the proposed deep learning architectures.

## Method validation

To validate the effectiveness of the proposed multi-output sliding window forecasting approach, the method was evaluated on the daily Rupiah to USD exchange rate dataset published by Bank Indonesia. The evaluation compared the performance of several forecasting models, like Multi-Layer Perceptron (MLP), Long Short-Term Memory (LSTM), Gated Recurrent Unit (GRU), and Vector Autoregression (VAR), under identical preprocessing, feature engineering, and training conditions, ensuring a fair and consistent comparison between deep learning and traditional statistical approaches.

The model outputs were then inverse-transformed from the normalized scale back to the original exchange rate values using the MinMaxScaler, ensuring that performance metrics reflected real-world currency units. Initially, the dataset was divided into training and testing sets with an 80:20 ratio to identify the most effective model architecture. Three evaluation metrics were employed: Mean Absolute Percentage Error (MAPE), Coefficient of Determination (R²), and Root Mean Square Error (RMSE), which captures the dispersion of prediction errors. Table 8 presents the comparative results of all tested models. The GRU model achieved the lowest MAPE (0.003709) and RMSE (74.816406) also highest R² (0.978758), indicating superior predictive accuracy and stability in capturing temporal patterns compared to LSTM and all MLP variants.

**Table 8:** Evaluation of the goodness and performance of the model.

No.	Type	RMSE	MAPE	R²
1	GRU	74.816406	0.003709	0.978758
2	LSTM	83.587332	0.004191	0.973485
3	MLP-Tuned2	135.739396	0.006439	0.930077
4	MLP-Tuned1	142.639107	0.006847	0.922788
5	MLP-Large	142.639107	0.006847	0.922788
6	MLP-Medium	190.383097	0.008235	0.862449
7	MLP-Small	224.003347	0.010174	0.809579

Modern models like GRU, LSTM, and tuned MLPs were compared against a simple VAR benchmark to evaluate predictive performance on the daily Rupiah to USD exchange rate dataset. The VAR model used a rolling 60-day window and dynamically selected the optimal lag order based on AIC, forecasting log returns of Selling Rate and Purchase Rate which were then exponentiated back to price levels. Despite its simplicity, VAR showed substantially higher RMSE (477.026056), MAPE (0.026740), and very low R² (0.144390) compared to GRU, indicating limited accuracy in capturing temporal patterns. This highlights that while VAR provides a straightforward baseline, modern deep learning models like GRU achieve superior predictive performance and stability.

To further evaluate the robustness of the best-performing GRU model, additional experiments were performed using 70:30 and 90:10 train-test splits. The results, summarized in Table 9, consistently confirmed the GRU model’s reliability and adaptability across different data proportions. The 70:30 configuration produced the most balanced and stable performance across all evaluation metrics, and therefore was selected as the final architecture for subsequent forecasting analysis.

**Table 9:** Performance of GRU model under different train-test split configurations.

Split	RMSE	MAPE	$R^{2}$
90:10	81.738249	0.004072	0.961236
80:20	62.154981	0.002906	0.985339
70:30	64.572612	0.003091	0.987501

**Fig. 1:**
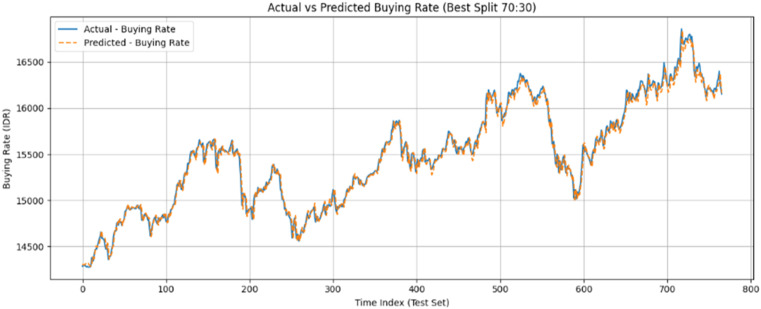
Comparison between actual and predicted exchange rates using the GRU model (buy rates) in 70:30 Architecture.

**Fig. 2:**
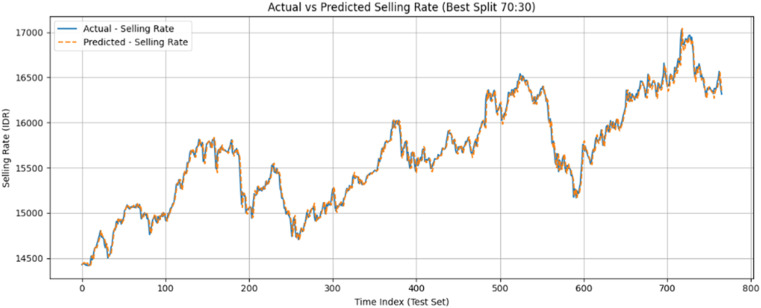
Comparison between actual and predicted exchange rates using the GRU model (sell rates) in 70:30 Architecture.

The predictive accuracy of the selected GRU (70:30) model was further illustrated through a visual comparison of actual versus predicted exchange rates. As shown in Figs. 1 and 2, the GRU model closely tracked the actual selling and buying rates over time, maintaining high accuracy even during periods of significant market volatility.

The final equation form of the GRU prediction model can be expressed as:

$$\hat{y}=0.211626232\cdot h+0.00877849\;$$

Where $\hat{y}$ is the predicted selling rate generated by the GRU model, and ℎ is the output of the last GRU unit at the final time step. This equation illustrates the transformation of the learned internal representation from historical sequences into a quantitative forecast in a single output dimension. The predicted values are then converted back to their original scale via inverse scaling before further interpretation and analysis.

To demonstrate the model’s applicability for forward-looking forecasting, the best-performing GRU (70:30) model was employed to predict exchange rates for the next seven days using a recursive, autoregressive strategy. Predictions incorporated cyclical time features (sin_day and cos_day) to maintain weekly seasonality. The results, shown in Table 10, were converted back to the original scale, illustrating realistic short-term projections.

**Table 10:** Predicted results of selling and buying rates for the next 1 week.

Day to	Selling Rate (Rp)	Purchase Rate (Rp)
1	16,322.02	16,163.43
2	16,250.57	16,098.19
3	16,162.80	16,011.54
4	16,059.93	15,914.15
5	15,954.28	15,816.93
6	15,856.65	15,723.66
7	15,768.79	15,636.96

These experimental results confirm that the proposed method, particularly when implemented with a GRU architecture using the 70:30 train-test configuration, is effective in short-term exchange rate prediction. The model demonstrates high accuracy, low prediction error, and consistent generalization performance across varying data partitions, outperforming both deep learning and traditional statistical baselines. This establishes the GRU-based multi-output sliding window approach as a reliable and robust framework for practical foreign exchange rate forecasting applications.

## Limitations

This method was developed and validated using historical exchange rate data from Bank Indonesia, focusing solely on the buying and selling rates of the Rupiah against the US Dollar. The method does not take into account external macroeconomic variables such as inflation, interest rates, or capital flows, which may influence exchange rate fluctuations. Its predictive capability has been demonstrated for short-term forecasting (seven days ahead) and may not generalize to long-term predictions or situations involving sudden economic or political shocks. Furthermore, the current model has only been directly compared to economic models such as VAR (Vector Autoregressive), which are typically used to account for macroeconomic variables more comprehensively. For future development, it is strongly recommended to compare this deep learning model with VARX (Vector Autoregressive with Exogenous variables). This approach would allow the integration of external variables such as inflation, interest rates, and capital flows, which could enhance predictive accuracy and extend the forecasting horizon in the face of more complex market fluctuations.

## Ethics statements

Not applicable. This study did not involve human participants, animal experiments, or data from social media platforms.

## Supplementary material *and/or* additional information [optional]

None

## CRediT authorship contribution statement

**Ihsan Fathoni Amri:** Conceptualization, Supervision, Writing – review & editing. **Novia Yunanita:** Software, Investigation, Data curation, Writing – original draft, Writing – review & editing. **Febi Anggun Lestari:** Methodology, Validation, Visualization. **Oktaviana Rahma Dhani:** Writing – original draft, Visualization.

## Declaration of competing interest

The authors declare that they have no known competing financial interests or personal relationships that could have appeared to influence the work reported in this paper.

## Data Availability

Data will be made available on request.
